# Insomnia Symptoms and Cardiovascular Disease among Older American Indians: The Native Elder Care Study

**DOI:** 10.1155/2011/964617

**Published:** 2011-12-19

**Authors:** Charumathi Sabanayagam, Anoop Shankar, Dedra Buchwald, R. Turner Goins

**Affiliations:** ^1^Department of Community Medicine, West Virginia University School of Medicine, Robert C. Byrd Health Sciences Center,1 Medical Center Drive, P.O. Box 9190, Morgantown, WV 26505, USA; ^2^Singapore Eye Research Institute, Singapore National Eye Centre, 11 Third Hospital Avenue #05-00, Singapore 168751; ^3^Department of Medicine, University of Washington, Seattle, WA 98195, USA; ^4^Center for Healthy Aging Research, School of Social and Behavioral Health Sciences, College of Public Health and Human Sciences, Oregon State University, Corvallis OR 97331, USA

## Abstract

*Background*. Cardiovascular disease (CVD) is the leading cause of death among American Indians. It is not known if symptoms of insomnia are associated with CVD in this population. *Methods*. We examined 449 American Indians aged ≥55 years from the Native Elder Care Study. The main outcome-of-interest was self-reported CVD. *Results*. Short sleep duration, daytime sleepiness, and difficulty falling asleep were positively associated with CVD after adjusting for demographic, lifestyle, and clinical risk factors. Compared with a sleep duration of 7 h, the multivariable odds ratio (OR) (95% confidence interval [CI]) of CVD among those with sleep duration ≤5 h was 2.89 (1.17–7.16). Similarly, the multivariable OR (95% CI) of CVD was 4.45 (1.85–10.72) and 2.60 (1.25–5.42) for daytime sleepiness >2 h and difficulty falling asleep often/always. *Conclusion*. Symptoms of insomnia including short sleep duration, daytime sleepiness, and difficulty falling asleep are independently associated with CVD in American Indians aged ≥55 years.

## 1. Introduction

Sleep complaints are common in older adults [1] and more than half of the older adults in a large population-based study had complaints related to inadequate sleep [2]. Recent studies have shown that sleep disturbances related to insomnia including short [3–5] or long [4, 5] sleep duration, daytime sleepiness [6], and difficulty falling asleep [3, 7] are associated with cardiovascular disease (CVD). Several prospective and cross-sectional studies have examined the association between sleep duration and CVD [3–5, 8–13]. However, there is limited information on the association between sleep duration and CVD in elderly populations [3, 9, 11]. Further, studies that examined the association between sleep disturbances including shorter or longer sleep duration, daytime sleepiness, difficulty falling asleep, and CVD among elderly adults were conducted in specific populations including women [9], men [14], non-Hispanic whites [3, 7, 11], or Japanese [11]. To our knowledge, no previous study has reported the association between insomnia symptoms and CVD in older American Indian adults. Although the multiracial Sleep Heart Health Study (SHHS) reported that sleep-disordered breathing was associated with heart failure, no subgroup analysis by race or ethnicity was provided in that report [15]. Among American Indians, CVD mortality rates equal or exceed national All Races rates [16] and CVD is the leading cause of death beginning at age 45 as opposed to age 65 in the general population [17]. Further, the prevalence of traditional CVD risk factors, including diabetes, hypertension, and dyslipidemia, is on the rise among American Indians [18]. However, it is not clear if insomnia symptoms contribute to the rising prevalence of CVD in this population. In this context, we examined the association between sleep duration, daytime sleepiness, and difficulty falling asleep and CVD in a population-based sample of American Indians.

## 2. Methods

The data for this study was collected as part of the Native Elder Care Study, a cross-sectional study of disability among older American Indians. Detailed selection of study population and methods has been reported previously [19, 20]. In brief, a random sample of 633 American Indians aged ≥55 years was drawn from a list of 1,430 individuals provided by tribal enrollment records. Of the 583 eligible participants, 505 participated in the baseline survey (86.6% response rate). After excluding those with missing information on sleep variables (*n* = 14) and other variables included in the multivariable model (*n* = 42), 449 subjects were included in the final analysis. Ethics approval was obtained from the tribe's institutional review board, Tribal Council, and Elder Council. This secondary data analysis was approved by the West Virginia University Institutional Review Board.

### 2.1. Assessment of CVD

The main outcome of interest was CVD defined as a physician diagnosis of heart attack, angina, heart failure, or stroke. This was determined by a “yes” response to the following four questions: “since age 55 has a doctor told you that you had angina, or heart attack, or congestive heart failure, or “stroke”?

### 2.2. Assessment of Exposure

Sleep duration, daytime sleepiness, and difficulty falling asleep were assessed from a questionnaire. Sleep duration for the 12-hour time period in night was assessed from the question “on average, how long do you sleep per night?” The response coded in hours was categorized into five groups for analysis: ≤5, 6, 7, 8, ≥9 hours. In addition to night time sleep duration, we also assessed 24-hour sleep duration from the question, “how many total hours do you sleep in an average 24-hour period?” The response categorized into the same five groups as night time sleep duration. Daytime sleepiness for the 12-hour daytime period was assessed from the question “How often do you fall asleep during the day against your will [17]?” As almost everyone in this elderly cohort reported feeling sleepy at least for an hour during the daytime, the response was categorized into three groups: 1, 2, >2 hours. Difficulty falling asleep in the night was assessed from the question “How often do you have difficulty falling asleep or staying asleep?” The response categorized into 3 groups: never, sometimes, often/always.

 Information on demographic, lifestyle, personal, and medical history was assessed from an interviewer-administered questionnaire. Education was recorded as the highest number of years of schooling completed and was categorized into below high school, high school, and above high school education. Current smokers were defined as those who answered affirmatively to the question “Do you currently smoke cigarettes?” Current alcohol drinking was defined as drinking alcohol everyday regardless of quantity. Physical activity was defined as engaging in physical activities such as running, swimming, aerobics, gardening, or walking for exercise. Symptoms of depression were assessed using the Center for Epidemiologic Studies depression Scale (CES-D) [21], a multidimensional screening instrument for major or clinical depression. In CES-D, each of the 20 response items is coded on a scale of 0–3 with the total scores ranging from 0–60. Depression was defined as a score of ≥16 on the CES-D scale. Presence of diabetes mellitus, hypertension, cancer, chronic pain, and back pain were ascertained by a “yes” response to the following five questions: “Since age 55 has a doctor told that you had diabetes, or high blood pressure, or cancer, or back pain, or chronic pain syndrome? Height was measured in centimeters using a measuring tape and weight in kilograms using a weighing scale. Body mass index (BMI) was calculated as weight in kilograms divided by the square of height in meters.

### 2.3. Statistical Analysis

We first performed descriptive statistics for all variables. All analyses were performed incorporating the sampling weights to account for the oversampling of aged people. We then examined the association between categories of sleep duration (7 h as the reference), daytime sleepiness (1 h as the reference), and difficulty falling asleep (never as the reference) and CVD in two logistic regression models separately for each sleep disturbance: (1) adjusted for age and sex, (2) additionally adjusted for education (<high school, high school, above high school), current smoking (absent, present), current alcohol consumption (absent, present), physical activity (absent, present), BMI (kg/m^2^), diabetes (absent, present), hypertension (absent, present), depression (absent, present), cancer (absent, present), chronic pain (absent, present), and back pain (absent, present). As the three sleep disturbances might be intercorrelated with each other, in a separate analysis, we included all three sleep variables simultaneously in the multivariable model and repeated the analysis. We performed tests for trend using the categories of sleep variables as ordinal variables in the corresponding multivariable model. All statistical analyses were performed using SAS version 9.1.

## 3. Results

**Figure 1:**
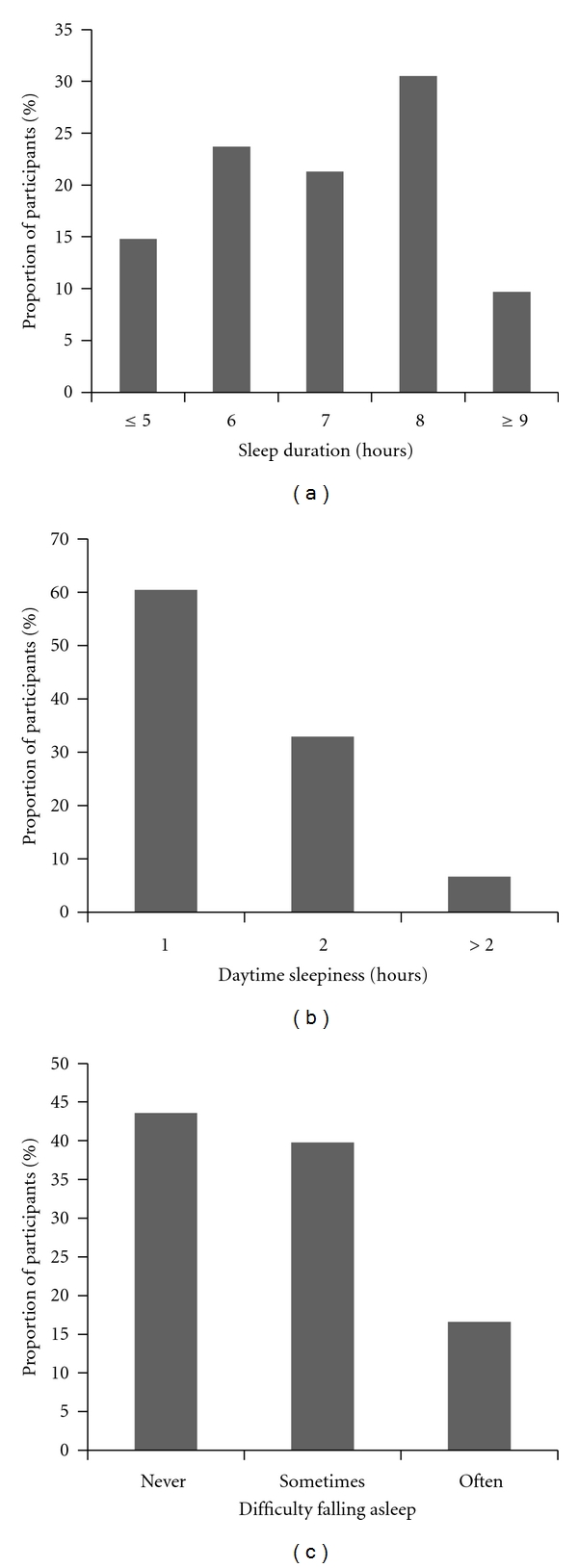
Prevalence of sleep disturbances in the study population.

The mean age of the study population was 65.9 y (SD = 8.5 y) and 62% were female. The prevalence of various comorbidities was high in this elderly population (diabetes = 40.9%, hypertension = 56.6%, depression = 13.8%, cancer = 9.5%, chronic pain = 11.2%, and back pain = 34.9%) (data not shown).  Table 1 shows the baseline characteristics of the study population by gender. Women were less likely to be current drinkers and more likely to report difficulty falling asleep than men. There was no significant difference (*P* = 0.9) in the reported average sleep duration between men and women (6.8 versus 6.9 h). The prevalence of each sleep disturbance is presented in Figure 1. 21.3% of the participants reported sleeping 7 h and 14.8% of the participants reported sleeping ≤5 h. 6.7% of the participants reported having daytime sleepiness >2 h and 16.6% of the participants had difficulty falling asleep often/always.

In univariate analysis, older age, below high school education, diabetes, hypertension, cancer, chronic pain, and back pain were associated with CVD; older age, current smoking, depression, chronic pain, and back pain were significantly associated with sleep duration ≤5 h (all *P* < 0.05).  Table 2 shows the relationship between the sleep disturbances and CVD after adjusting for potential confounders. The prevalence of CVD was highest among those who reported sleeping ≤5 h and lowest among those who reported sleeping 7 h. There was a significant positive association between sleep duration ≤5 h and CVD in both the age, sex-adjusted, and the multivariable model additionally adjusted for education, current smoking, current alcohol drinking, physical activity, diabetes mellitus, hypertension, depression, cancer, chronic pain, and back pain. The prevalence of CVD increased with increasing categories of daytime sleepiness (*P*-trend < 0.0001) and daytime sleepiness >2 h was significantly associated with CVD in both the models. Similarly, the prevalence of CVD increased with increasing categories of difficulty falling asleep (*P*-trend = 0.006) and the association between difficulty falling asleep often/always was significant in both the models.

In a supplementary analysis, when we included all three sleep disturbances simultaneously in the multivariable model, although all 3 sleep disturbances showed a positive magnitude of association with CVD, only the association of daytime sleepiness >2 h with CVD was statistically significant (OR [95%] = 4.16 [1.67–10.32] for daytime sleepiness >2 h) and not for sleep duration or difficulty falling asleep (OR [95%] = 1.90 [0.68–5.34] for sleep duration ≤5 h and 2.00 [0.84–4.76] for difficulty falling asleep). In a second supplementary analysis, when we assessed the association between 24 h sleep duration and CVD, the pattern of association was similar to that of night time sleep duration. Compared with a sleep duration of 7 h, the multivariable odds ratio (OR) (95% confidence interval [CI]) of CVD was 5.37 (1.94–14.91) for sleep duration ≤5 h and 2.26 (0.92–5.54) for >8 h.

## 4. Discussion

In a population-based study of American Indian adults aged ≥55 years, short sleep duration, daytime sleepiness, and difficulty falling asleep were significantly associated with CVD independent of age, sex, education, current smoking, current alcohol consumption, physical activity, BMI, diabetes, hypertension, depression, cancer, chronic pain, and back pain. To our knowledge, this is the first study to separately report the association between various sleep characteristics and CVD among American Indians. Our findings of associations between sleep duration, daytime sleepiness, difficulty falling asleep, and CVD are consistent with previous studies that reported a similar association in older Hispanic and non-Hispanic whites and African Americans [3, 4, 6, 7] and extend the association to middle-aged and older adults in the American Indian population.

In the current study, sleep duration ≤5 h was associated with CVD. Similar to our report, sleep duration <6 h was associated with CVD among 1506 men and women aged ≥55 years who participated in the 2003 Sleep in America poll [3] and sleep duration <7.5 h was associated with incident CVD among 1255 elderly adults with hypertension in Japan [13]. Few studies have documented a “*U*” shaped association between sleep duration and CVD [4, 5, 8] and CVD mortality [22, 23]. A recent meta-analysis including 15 prospective studies documented a positive association between both short and long durations of sleep and CVD [8]. Both short and long durations of sleep were associated with CVD among adults aged >60 years in the National Health Interview Survey 2005 [4] and with coronary heart disease among female health professionals aged 45–65 years in the Nurses' Health study [5]. In contrast to our study, few studies have documented an association between long duration of sleep and CVD [9, 12]. Sleep duration ≥9 hours was shown to be associated with CVD among a large cohort of postmenopausal women in the Women's Health Initiative [9] and 3,430 middle-aged Chinese adults in Taiwan [12]. 

In the current study, daytime sleepiness and difficulty falling asleep were associated with CVD similar to previous reports. In the Cardiovascular Health Study including 5,888 whites and African Americans [6], daytime sleepiness was associated with CVD, and in an earlier study, difficulty falling asleep was associated with angina in the same population [7]. Daytime sleepiness was associated with stroke among 7,844 adults who participated in the NHANES-I followup study in the US and a large cohort of men aged 55–69 years in the UK [14].

 An association between symptoms of insomnia and CVD is biologically plausible. Short sleep duration, daytime sleepiness, and difficulty falling asleep through metabolic and endocrine dysfunctions lead to impaired glucose tolerance, increased insulin resistance, enhanced sympathetic activity, and increased blood pressure with resultant CVD [24, 25]. Studies have also documented an association between insomnia symptoms and risk factors of CVD including diabetes [3, 26], hypertension [27], obesity [28] dyslipidemia [29], and metabolic syndrome [30].

The strengths of the current study include its population-based nature and the availability of data on potential confounders. Our study has several limitations. First, sleep habits were self-reported and the associated measurement error might have resulted in a nondifferential misclassification of exposure. Second, we assessed daytime sleepiness using a single question rather than the standard Epworth Sleepiness Score (ESS). However, daytime sleepiness assessed using a single question is comparable to that measured using ESS [31] and several epidemiological studies have assessed daytime sleepiness using a single question similar to ours [32, 33]. Third, it is possible that our results may be biased by residual confounding from unmeasured variables including sleep disordered breathing (SDB) that play a role in CVD. Fourth, although the sample size was adequate to detect an overall association, it was insufficient to perform subgroup analysis and to explore effect modifications. Fifth, because of the cross-sectional nature of the study, we cannot infer causal associations.

In conclusion, our study shows that short sleep duration, daytime sleepiness, and difficulty falling asleep were associated with CVD among American Indians. If confirmed by future prospective studies, our findings may have important implications for targeting sleep habits in intervention programs intended to reduce the burden of CVD among American Indians.

## Figures and Tables

**Table 1:** Baseline characteristics of the study population by gender.

Characteristics	Men (*n* = 163)	Women (*n* = 286)	*P*-value
Age (years)	64.9 (8.0)	66.4 (8.7)	0.06
Education categories (%)			0.5
Below high school	29.1	33.2	
High school	30.9	32.2	
Above high school	40.0	34.6	
Current smoker (%)	21.8	27.7	0.2
Current drinker (%)	19.6	8.1	0.0003
Moderate physical activity (%)	66.8	57.6	0.05
Body mass index (kg/m^2^)	30.0 (5.4)	29.9 (6.7)	0.9
Diabetes mellitus (%)	36.8	43.5	0.2
Hypertension (%)	54.8	57.7	0.5
Cancer (%)	10.6	15.9	0.1
Depression (%)	8.2	10.4	0.4
Chronic pain (%)	11.4	11.1	0.9
Back pain (%)	30.3	37.7	0.1
Night sleep duration (hours)	6.8 (1.7)	6.9 (1.6)	0.9
Daytime sleepiness (hours)	1.6 (0.7)	1.4 (0.6)	0.05
Difficulty falling asleep (hours)	1.7 (0.8)	1.9 (0.9)	0.003

*Data presented are percentages or mean values (standard deviation), as appropriate for the variable.

**Table 2:** Association between sleep variables and cardiovascular disease.

Sleep variables	No at risk* (*n* = 449)	Weighted prevalence of CVD, %	Age, sex adjusted OR (95% CI)	Multivariable adjusted OR (95% CI)^†^
Sleep duration, h				
≤5	58	29.2	3.51 (1.55–7.95)	2.89 (1.17–7.16)
6	101	17.6	1.55 (0.71–3.42)	1.32 (0.56–3.09)
7	98	12.6	1 (referent)	1 (referent)
8	145	22.1	1.85 (0.89–3.85)	1.67 (0.76–3.66)
≥9	47	18.5	1.46 (0.54–3.93)	1.15 (0.38–3.53)
Frequency of daytime sleepiness, h				
1	253	14.3	1 (referent)	1 (referent)
2	161	22.6	1.53 (0.91–2.57)	1.33 (0.76–2.34)
>2	35	54.6	6.06 (2.71–13.52)	4.45 (1.85–10.72)
*P*-trend			<0.0001	0.003
Difficulty falling asleep				
Never	200	14.8	1 (referent)	1 (referent)
Sometimes	178	20.0	1.60 (0.92–2.78)	1.23 (0.68–2.25)
Often or always	71	31.9	3.39 (1.76–6.53)	2.60 (1.25–5.42)
*P*-trend			0.0004	0.02

Abbreviations: CI: confidence interval; OR: odds ratio.

*Unweighted sample size

^†^Adjusted for age (years), sex (men, women), education (<high school, high school, >high school), current smoking (absent, present), current alcohol consumption (absent, present), physical activity (absent, present), body mass index (kg/m^2^), diabetes (absent, present), hypertension (absent, present), depression (absent, present), cancer (absent, present), chronic pain (absent, present) and back pain (absent, present).
